# Supplementary material for: Comparison of Machine Learning Techniques for Prediction of Hospitalization in Heart Failure Patients
Source: J Clin Med. 2019 Aug 24;8(9):1298. doi: 10.3390/jcm8091298 (PMC6780582; doi:10.3390/jcm8091298)
Supplement: Supplementary file 1 [file jcm-08-01298-s001.pdf]

Supplementary Material. Overview of the Machine Learning Techniques used in literature along with their main features. Rearranged from the work of (IZSTO et al., 2017)

**MLT**	**Label**	**Output** (#)	**Output** (type)	**Sample size**	**Effect**	**Predict**
ANN (Artificial Neural Networks)	Both	Multiple	Any	Medium-high	No	No
KNN (K-nearest neighbor)	Both	Single	Any	Small	No	Yes
EA (Evolutionary Algorithm)	Supervised	Multiple	Any	Small	No	Yes
GMDH (Group Method of Data Handling)	Supervised	Multiple	Continuous	High	No	No
IBL (Instance-based learning)	Supervised	Multiple	Any	Medium	No	No
SVM (Support-Vector Machine)	Supervised	Multiple	Any	High	No	No
Bayesian Networks	Supervised	Multiple	Categorical	Medium-high	Yes	Yes
Hidden Markov Model	Supervised	Multiple	Categorical	Medium	Yes	No
AODE (Average one- dependence estimator)	Supervised	Single	Categorical, Binary	Small	No	No
Kriging (Gaussian process regression)	Supervised	Single	Continuous, Time series	Small	Yes	No
ANOVA (Analysis Of Variance)	Supervised	Single	Continuous	Small	No	No
Fischer's Linear Discriminant	Supervised	Single	Binary	Small	No	No
Logistic Regression	Supervised	Single	Binary	Small	Yes	Yes
Multinomial Logistic Regression	Supervised	Single	Categorical	Small	Yes	Yes
Naive Bayes Classifier	Supervised	Single	Any	Small	Yes	No
LMT (Logistic Model Tree)	Supervised	Single	Binary	High	Yes	No
Random Forest	Supervised	Single	Categorical	Small	No	No
Quadratic Classifier	Supervised	Single	Binary	Small	No	No
Decision Tree	Supervised	Single	Categorical	Small	No	Yes
BART	Supervised	Single	Categorical	Small	Yes	Yes
Vector Quantization	Supervised	Single	Time series	High	No	No
Moment methods and EM algorithms	Supervised	Single	Any	Small	Yes	Yes
CWM (Cluster Weighted modeling)	Supervised	Single	Time series	Small	No	No

MRF (Markov Random Fields)	Supervised	Multiple	Categorical	Medium	No	No
ILP (Inductive Logic Programming)	Supervised	Multiple	General objects	Small	No	No
Hierarchical clustering	Unsupervised	Multiple	Continuous	\	No	No
OPTICS, DBSCAN	Unsupervised	Multiple	Continuous, Time series	Small	No	No
K-means	Unsupervised	Multiple	Continuous	Small-Medium	No	No
Self-Organizing Maps (SOM)	Unsupervised	Multiple	Continuous	Small	No	No

IZSTO, Ru, G., Crescio, M. I., Ingravalle, F., Maurella, C., UBESP, … Lorenzoni, G. (2017). Machine Learning Techniques applied in risk assessment related to food safety. *EFSA Supporting Publications*, *14*(7), 1254E.
